# Assessment of Vitamin D Metabolism in Patients with Cushing’s Disease in Response to 150,000 IU Cholecalciferol Treatment

**DOI:** 10.3390/nu13124329

**Published:** 2021-11-30

**Authors:** Alexandra Povaliaeva, Viktor Bogdanov, Ekaterina Pigarova, Artem Zhukov, Larisa Dzeranova, Zhanna Belaya, Liudmila Rozhinskaya, Galina Mel’nichenko, Natalia Mokrysheva

**Affiliations:** Endocrinology Research Centre, 117292 Moscow, Russia; bogdanov.viktor@endocrincentr.ru (V.B.); pigarova.ekaterina@endocrincentr.ru (E.P.); jukov.artem@endocrincentr.ru (A.Z.); dzeranova.larisa@endocrincentr.ru (L.D.); belaya.zhanna@endocrincentr.ru (Z.B.); rozhinskaya.ludmila@endocrincentr.ru (L.R.); melnichenko.galina@endocrincentr.ru (G.M.); mokrisheva.natalia@endocrincentr.ru (N.M.)

**Keywords:** vitamin D, pituitary ACTH hypersecretion, cholecalciferol, vitamin D-binding protein

## Abstract

In this study we aimed to assess vitamin D metabolism in patients with Cushing’s disease (CD) compared to healthy individuals in the setting of bolus cholecalciferol treatment. The study group included 30 adults with active CD and the control group included 30 apparently healthy adults with similar age, sex and BMI. All participants received a single dose (150,000 IU) of cholecalciferol aqueous solution orally. Laboratory assessments including serum vitamin D metabolites (25(OH)D_3_, 25(OH)D_2_, 1,25(OH)_2_D_3_, 3-epi-25(OH)D_3_ and 24,25(OH)_2_D_3_), free 25(OH)D, vitamin D-binding protein (DBP) and parathyroid hormone (PTH) as well as serum and urine biochemical parameters were performed before the intake and on Days 1, 3 and 7 after the administration. All data were analyzed with non-parametric statistics. Patients with CD had similar to healthy controls 25(OH)D_3_ levels (*p* > 0.05) and higher 25(OH)D_3_/24,25(OH)_2_D_3_ ratios (*p* < 0.05) throughout the study. They also had lower baseline free 25(OH)D levels (*p* < 0.05) despite similar DBP levels (*p* > 0.05) and lower albumin levels (*p* < 0.05); 24-h urinary free cortisol showed significant correlation with baseline 25(OH)D_3_/24,25(OH)_2_D_3_ ratio (r = 0.36, *p* < 0.05). The increase in 25(OH)D_3_ after cholecalciferol intake was similar in obese and non-obese states and lacked correlation with BMI (*p* > 0.05) among patients with CD, as opposed to the control group. Overall, patients with CD have a consistently higher 25(OH)D_3_/24,25(OH)_2_D_3_ ratio, which is indicative of a decrease in 24-hydroxylase activity. This altered activity of the principal vitamin D catabolism might influence the effectiveness of cholecalciferol treatment. The observed difference in baseline free 25(OH)D levels is not entirely clear and requires further study.

## 1. Introduction

Cushing’s disease (CD) is one of the disorders associated with endogenous hypercortisolism and is caused by adrenocorticotropic hormone (ACTH) hyperproduction originating from pituitary adenoma [1]. Skeletal fragility is a frequent complication of endogenous hypercortisolism, and fragility fractures may be the presenting clinical feature of disease. The prevalence of osteoporosis in endogenous hypercortisolism as assessed by dual-energy X-ray absorptiometry (DXA) or incidence of fragility fractures has been reported to be up to 50%. Osteoporosis in CD patients has a complex multifactorial pathogenesis, characterized by a low bone turnover and severe suppression of bone formation [2]. Exogenous glucocorticoids are used in the treatment of a wide range of diseases and it is estimated that 1–2% of the population is receiving long-term glucocorticoid therapy. As a consequence, glucocorticoid-induced osteoporosis is the most common secondary cause of osteoporosis [3].

Native vitamin D (in particular D_3_, or cholecalciferol) and its active metabolites (such as alfacalcidol) are universally considered as the essential components of the osteoporosis management [4,5]. The search for the optimal treatment of bone complications during chronic exposure to glucocorticoid excess provoked the investigation of vitamin D metabolism in this state. Early studies on this topic were focused predominantly on the general vitamin D status (assessed as 25(OH)D level) and on the levels of the active vitamin D metabolite (1,25(OH)_2_D). These studies showed inconsistent results, reporting that the chronic excess of glucocorticoids decreased [6,7,8,9], increased [10,11,12] or did not change [13,14,15] the levels of 25(OH)D or 1,25(OH)_2_D. A likely reason for such inconsistency might have been the high heterogeneity of the studied groups. Some of these studies were performed in humans [6,7,9,10,11,12,13,15] and some in animal models [8,14], and only several of them included subjects with specifically endogenous hypercortisolism [10,12,14,15]. Only two studies assessed both the levels of the active (1,25(OH)_2_D) and the inactive (24,25(OH)_2_D) vitamin D metabolites in endogenous hypercortisolism. One of them lacked control group and reported low-normal 24,25(OH)_2_D levels in patients with Cushing’s syndrome [10]. The second study by Corbee et al. reported similar circulating concentrations of 25(OH)D, 1,25(OH)_2_D and 24,25(OH)_2_D in studied groups of dogs regardless of either the presence of CD or hypophysectomy status [14].

Several experimental studies were performed to evaluate the impact of glucocorticoid excess on the enzymes involved in vitamin D metabolism. In mouse kidney glucocorticoid treatment increased 24-hydroxylase expression [16] and 24-hydroxylase activity [17]. An increased expression of 24-hydroxylase was also shown in rat osteoblastic and pig renal cell cultures treated with 1,25(OH)_2_D [18]. Dhawan and Christakos showed that 1,25(OH)_2_D-induced transcription of 24-hydroxylase was glucocorticoid receptor-dependent [19]. However, some works showed conflicting results. In particular, the steroid and xenobiotic receptor (SXR) which is activated by glucocorticoids [20], repressed 24-hydroxylase expression in human liver and intestine in work by Zhou et al. [21]. Lower 24-hydroxylase expression was observed in the brain and myocardium of glucocorticoid-treated rats [22] as well as in human osteosarcoma cells and human osteoblasts [23].

Nevertheless, based on experimental data, it has been suggested that the acceleration of 25(OH)D catabolism in the presence of glucocorticoid excess may predispose to vitamin D deficiency. Yet, relatively recent meta-analysis of the studies assessing 25(OH)D levels in chronic glucocorticoid users showed that serum 25(OH)D levels in these patients were suboptimal and lower than in healthy controls, but similar to steroid-naive disease controls [24]. 

Glucocorticoids also affect calcium and phosphorus homeostasis. In particular, they were shown to reduce gastrointestinal absorption by antagonizing vitamin D action (reducing the expression of genes for proteins involved in calcium transport—epithelial Ca channel TRPV6 and calcium-binding protein calbindin-D9K) [25]. Glucocorticoids increased fractional calcium excretion due to mineralocorticoid receptor-mediated action on epithelial sodium channels [26]. Hypercalciuria is highly prevalent in people with CD [27]. These effects might result in a negative calcium balance, although plasma ionized calcium was normal in people and dogs with hypercortisolism compared to control subjects [12,28]. Glucocorticoids also reduced tubular phosphate reabsorption by inhibiting tubular expression of the sodium gradient-dependent phosphate transporter, and induced phosphaturia [29], which was accompanied by phosphate lowering in humans [12].

Overall, current data on vitamin D status in hypercortisolism are conflicting and need clarification. In particular, clinical data on the state of vitamin D metabolism in the state of glucocorticoids excess are quite scarce. Studies were very heterogeneous in design, some lacked a control group, and the absolute majority of the studies were performed before the introduction of vitamin D measurement standardization [30]. Nevertheless, determining the optimal vitamin D treatment regimen in these high-risk patients is fairly relevant.

The aim of this study was to assess vitamin D metabolism in patients with CD compared to healthy individuals particularly in the setting of cholecalciferol treatment.

## 2. Materials and Methods

### 2.1. Study Population and Design

The study group included 30 adult patients with CD admitted for inpatient treatment at a tertiary pituitary center. Diagnosis of CD was established in accordance with the federal guidelines [31]. All patients were confirmed to be positive for endogenous hypercortisolism in at least two of the following tests: 24-h urine free cortisol (UFC) greater than the normal range for the assay and/or serum cortisol > 50 nmol/L after the 1-mg overnight dexamethasone suppression test and/or late-night salivary cortisol greater than 9.4 nmol/L). All patients also had morning ACTH ≥ 10 pg/mL and pituitary adenoma ≥ 6 mm identified by magnetic resonance imaging (MRI) or a positive for CD bilateral inferior petrosal sinus sampling (BIPSS). MRI was performed using a GE Optima MR450w 1.5T with Gadolinium (Boston, MA, USA). BIPSS was performed according to the standard procedure described elsewhere [32,33].

The control group included 30 apparently healthy adult individuals recruited from the staff and the faculty of the facility. 

Inclusion criteria were age from 18 to 60 for both groups and the presence of the disease activity for the study group (defined as the presence of endogenous hypercortisolism at the time of participation in the study). Exclusion criteria for both groups were: vitamin D supplementation for 3 months prior to the study; severe obesity (body mass index (BMI) ≥ 35 kg/m^2^); pregnancy; the presence of granulomatous disease, malabsorption syndrome, liver failure; decreased GFR (less than 60 mL/min per 1.73 m^2^); severe hypercalcemia (total serum calcium > 3.0 mmol/L); allergic reactions to vitamin D medications; 25(OH)D level more than 60 ng/mL (determined by immunochemiluminescence analysis). All patients were recruited in the period from October 2019 to April 2021. The study protocol (ClinicalTrials.gov Identifier: NCT04844164) was approved by the Ethics Committee of Endocrinology Research Centre, Moscow, Russia on 10 April 2019 (abstract of record No. 6), all patients signed informed consent to participate in the study.

All participants received standard therapeutic dose (150,000 IU) of an aqueous solution of cholecalciferol (Aquadetrim^®^, Medana Pharma S.A., Sieradz, Poland) orally as a single dose [34]. Blood and urine samples were obtained before the intake as well as on days 1, 3 and 7 after administration; time points of sample collection were determined based on the authors’ previous work evaluating changes in 25(OH)D levels after a therapeutic dose of cholecalciferol [35]. The assessment included serum biochemical parameters (total calcium, albumin, phosphorus, creatinine, magnesium), parathyroid hormone (PTH), vitamin D-binding protein (DBP), vitamin D metabolites (25(OH)D_3_, 25(OH)D_2_, 1,25(OH)_2_D_3_, 3-epi-25(OH)D_3_ and 24,25(OH)_2_D_3_), free 25(OH)D and urine biochemical parameters (calcium- and phosphorus-creatinine ratios in spot urine).

### 2.2. Socio–Demographic and Anthropometric Data Collection

At the baseline visit, patients underwent a questionnaire aimed to assess their lifestyle: the presence of unhealthy habits, physical activity level, balanced diet (consumption of dairy products, meat, coffee, soft drinks), exposure to ultraviolet (UV) radiation (solarium and sunscreen usage, traveling south and the number of daytime walks in the sunny weather in the 3 months preceding study participation). Smoking status was classified as current smoker, former smoker and non-smoker; current and former smokers were collectively referred to as total smokers. A unit of alcohol was defined as a glass of wine, a bottle of beer or a shot of spirits, approximating 10–12 g ethanol. Serving of dairy products was defined as 100 g of cottage cheese, 200 mL of milk, 125 g of yogurt or 30 g of cheese. Patients’ weight was measured in light indoor clothing with a medical scale to the nearest 100 g, and their height with a wall-mounted stadiometer to the nearest centimeter. BMI was calculated as weight in kilograms divided by height in meters squared.

### 2.3. Laboratory Measurements

Morning ACTH (reference range 7–66 pg/mL), serum cortisol after a low-dose dexamethasone suppression test (cutoff value for suppression, 50 nmol/L [36]), late-night salivary cortisol (reference range 0.5–9.4 nmol/L [37]) were assayed by electrochemiluminescence assay using a Cobas 6000 Module e601 (Roche, Rotkreuz, Switzerland). The 24-h UFC (reference range 60–413 nmol/24 h) was measured by an immunochemiluminescence assay (extraction with diethyl ether) on a Vitros ECiQ (Ortho Clinical Diagnostics, Raritan, NJ, USA).

Total 25(OH)D levels (25(OH)D_2_ + 25(OH)D_3_; reference range 30–100 ng/mL) at the baseline visit were determined by the immunochemiluminescence analysis (Liaison, DiaSorin, Saluggia, Italy). PTH levels were evaluated by the electrochemiluminescence immunoassay (ELECSYS, Roche, Basel, Switzerland; reference range for this and subsequent laboratory parameters are given in the Results section for easier reading). Biochemical parameters of blood serum and urine were assessed by the ARCHITECT c8000 analyzer (Abbott, Chicago, IL, USA) using reagents from the same manufacturer according to the standard methods. Serum DBP and free 25(OH)D levels were measured by enzyme-linked immunosorbent assay (ELISA) using commercial kits. The assay used for free 25(OH)D levels assessment (DIAsource, ImmunoAssays S.A., Ottignies-Louvain-la-Neuve, Belgium) has <6.2% intra- and inter-assay coefficient of variation (CV) at levels 5.8–9.6 pg/mL. The assay used for DBP levels assessment (Assaypro, St Charles, MO, USA) has 6.2% average intra-assay CV and 9.9% average inter-assay CV.

The levels of vitamin D metabolites (25(OH)D_3_, 25(OH)D_2_, 1,25(OH)2D_3_, 3-epi-25(OH)D_3_ and 24,25(OH)_2_D_3_) in serum were determined by ultra-high performance liquid chromatography in combination with tandem mass spectrometry (UPLC-MS/MS) using an in-house developed method, described earlier [38]. With this technique, the laboratory participates in DEQAS quality assurance program (lab code 2388) and the results fall within the target range for the analysis of 25(OH)D and 1,25(OH)_2_D metabolites in human serum (Supporting Information, Figures S1 and S2). All UPLC-MS/MS measurements were made after the first successful completion (5/5 samples within the target range) of the DEQAS distributions for both analytes simultaneously. Each batch contained control samples (analytes in blank serum) with both high and low analyte concentrations. The samples were barcoded and randomized prior to the measurements to eliminate analyst-related errors.

Serum samples (3 aliquots) collected at each visit were either transferred directly to the laboratory for biochemical analyzes, total 25(OH)D and PTH measurement (1 aliquot) or were stored at −80 °C avoiding repeated freeze-thaw cycles for measurement of DBP, free 25(OH)D and vitamin D metabolites at a later date (2 aliquots).

Albumin-adjusted serum calcium levels were calculated using the formula [39]: total plasma calcium (mmol/L) = measured total plasma calcium (mmol/L) + 0.02 × (40 − measured plasma albumin (g/L)).

Baseline free 25(OH)D levels were also calculated using the formula introduced by Bikle et al. [40,41]. The affinity constant for 25(OH)D and albumin binding (Kalb) used for the calculation was equal 6 × 10^5^ M^−1^, and affinity constant for 25(OH)D and DBP binding (KDBP) was equal 7 × 10^8^ M^−1^.

$$\mathrm{Free}\;25(\mathrm{OH})D=\frac{\mathrm{total}\;25\left(\mathrm{OH}\right)D}{1+K_{\mathrm{alb}}*\mathrm{albumin}+\mathrm{KDBP}*\mathrm{DBP}}$$

### 2.4. Statistical Analysis

Statistical analysis was performed using Statistica version 13.0 (StatSoft, Tulsa, OK, USA). All data were analyzed with non-parametric statistics and expressed as median [interquartile range] unless otherwise specified. Mann-Whitney U-test and Fisher’s exact two-tailed test were used for comparisons between two groups. Friedman ANOVA was performed to evaluate changes in indices throughout the study and pairwise comparisons using Wilcoxon test with adjustment for multiple comparisons (Bonferroni) were also made if the Friedman ANOVA was significant. Spearman rank correlation method was used to obtain correlation coefficients among indices. A *p*-value of less than 0.05 was considered statistically significant. When adjusting for multiple comparisons, a *p*-value greater than the significance threshold, but less than 0.05 was considered as a trend towards statistical significance. 

## 3. Results

The groups were similar in terms of age, sex and BMI (*p* > 0.05). Both groups consisted predominantly of young and middle-aged women and the majority of patients were overweight or moderately obese (Table 1). Patients from the study group presented with lower screening levels of total 25(OH)D (*p* < 0.05).

The features of the underlying disease course in the study group are listed in Table 2. 15 patients (50%) had diabetes mellitus with an almost compensated state at the time of participation in the study, and 7 patients (23%) reported a history of low-energy fractures.

The groups did not differ significantly in the reported smoking status, the level of daily physical activity, dietary habits and UV exposure (*p* > 0.05) and although there was a slight difference in alcohol consumption (*p* < 0.05), the absolute values were minor in both groups (Table 3).

### 3.1. Baseline Laboratory Evaluation

Detailed results of laboratory studies are presented in Table 4 and Table 5.

Patients with CD had several alterations in biochemical parameters, in particular, lower baseline serum creatinine and albumin levels, while magnesium levels were higher than in the control group (*p* < 0.05). They also had higher levels of urine phosphorus-creatinine ratio (*p* < 0.05). The rest of the studied biochemical parameters did not show significant difference between the groups (*p* > 0.05). 3 patients (10%) from the study group and 5 patients (17%) from the control group had secondary hyperparathyroidism, one patient with CD (3%) was diagnosed with mild primary hyperparathyroidism.

As for the assessment of vitamin D metabolism, unexpectedly the levels of 25(OH)D_3_ occurred to be equal in the groups (*p* > 0.05), with only two patients (7%) from the study group and one patient (3%) from the control group having sufficient vitamin D levels, according to the Endocrine Society and the Russian Association of Endocrinologists guidelines (≥30 ng/mL [34,42]). The levels of the active vitamin D metabolite—1,25(OH)_2_D_3_—were equal between the groups as well (*p* > 0.05), whereas the levels of 3-epi-25(OH)D_3_ and 24,25(OH)_2_D_3_ were lower in CD patients. Further calculation of 25(OH)D_3_/24,25(OH)_2_D_3_ and 25(OH)D_3_/1,25(OH)_2_D_3_ ratios corresponded to the observed levels of metabolites: 25(OH)D_3_/24,25(OH)_2_D_3_ ratio was higher in the study group (*p* < 0.05) assuming lower 24-hydroxylase activity and 25(OH)D_3_/1,25(OH)_2_D_3_ ratio was equal between the groups (*p* > 0.05). 

Levels of free 25(OH)D were lower in CD patients (*p* < 0.05) and the levels of DBP did not differ between the groups (*p* > 0.05). Although calculated free 25(OH)D showed prominent positive correlation with the measured free 25(OH)D in both groups (r = 0.63 in the study group, r = 0.87 in the control group, *p* < 0.05), the association appeared to be weaker in the study group. In the control group, DBP levels correlated with both measured and calculated 25(OH)D levels (r = −0.48, *p* < 0.05 and r = −0.69, *p* < 0.05 respectively), while in patients with CD there was no association with measured free 25(OH)D levels (r = 0.04, *p* > 0.05 and r = −0.50, *p* < 0.05 respectively).

Correlation with 24-h UFC in CD patients was observed for serum albumin level (r = −0.37, *p* < 0.05) and urine calcium-creatinine ratio (r = 0.51, *p* < 0.05) among assessed biochemical parameters, and only with 25(OH)D_3_/24,25(OH)_2_D_3_ ratio among the parameters of vitamin D metabolism (r = 0.36, *p* < 0.05).

### 3.2. Laboratory Evaluation after the Intake of Cholecalciferol

All patients from the study group and 28 patients (93%) from the control group completed the study. 

The observed baseline differences in biochemical parameters mostly preserved during the follow-up. In the study group there was an increase in serum phosphorus levels by Day 1 (*p* = 0.006) and a tendency to an increase in the urine phosphorus-creatinine ratio by Day 7 (*p* = 0.02). Patients from the control group showed a clinically insignificant increase in serum creatinine levels by Day 1 (*p* = 0.002) and a non-significant trend towards an increase in serum total and albumin-adjusted calcium (*p* = 0.01 for both measurements). No change in PTH levels was observed in patients with CD during the follow-up (*p* > 0.05), while in the control group there was a tendency for PTH to decrease by Day 3 (*p* = 0.02). There were no new cases of hypercalcemia in both groups during the follow-up. One patient from the study group and one patient from the control group had persistently increased urine calcium-creatinine ratio throughout the study. Four patients from the study group (13%) and none from the control group developed hypercalciuria during the follow-up, however these patients had no clinical manifestations during the observation period.

By Day 7, 25 patients (83%) from the study group and 22 patients (79%) reached sufficient 25(OH)D_3_ levels (≥30 ng/mL). Levels of 25(OH)D_3_ continued to increase by Day 3 in both groups (*p* < 0.001), after which tended to decrease in the study group (*p* = 0.01) and remained stable in the control group (*p* = 0.65). The increase in 25(OH)D_3_ after cholecalciferol intake was equal between the groups (18.5 [15.9; 22.5] ng/mL in the study group vs. 16.6 [13.1; 19.8] ng/mL in the control group, *p* > 0.05). In the presence of obesity, Δ25(OH)D_3_ was higher in the CD patients than in the control group (18.3 [14.2; 23.0] vs. 12.1 [10.0; 13.1] ng/mL, *p* < 0.05), while in non-obese patients no difference was observed (*p* > 0.05).

**Figure 1:**
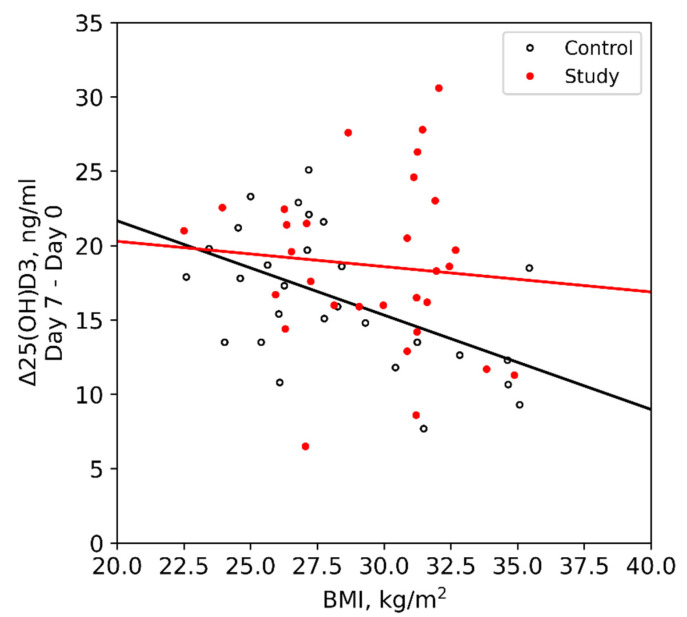
Relationship between Δ25(OH)D_3_ and BMI in groups.

Obese and non-obese patients with CD had equal Δ25(OH)D_3_ (18.3 [14.2; 23.0] vs. 19.6 [16.0; 21.5] ng/mL, *p* > 0.05), while in the control group it was significantly lower in obese patients (12.1 [10.0; 13.1] vs. 18.3 [15.3; 21.4] ng/mL, *p* < 0.05). BMI showed significant correlation with Δ25(OH)D_3_ only in the control group (r = −0.47, *p* < 0.05), while in CD patients there was no such association (r = −0.06, *p* > 0.05) (Figure 1).

**Figure 2:**
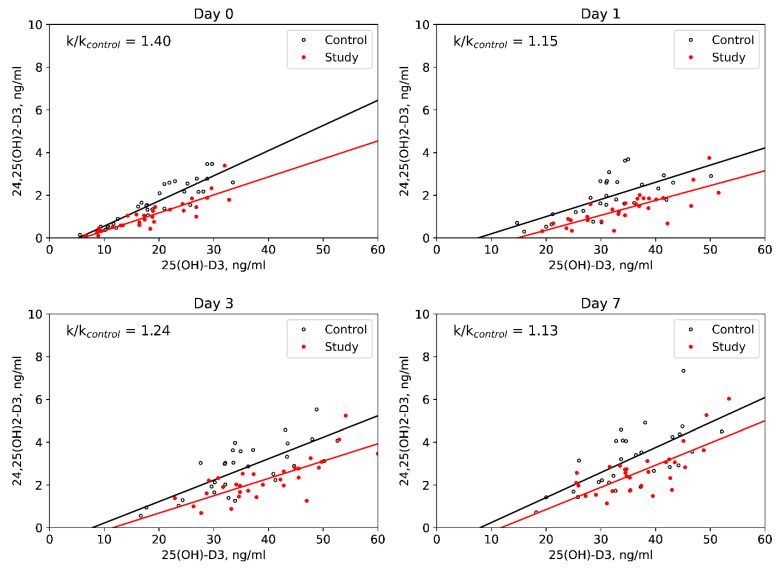
Dynamic evaluation of 25(OH)D_3_/24,25(OH)_2_D_3_ ratios in groups.

1,25(OH)_2_D_3_ levels increased in CD patients by Day 1 and were stable during the follow-up in the control group. The rest of the studied parameters of vitamin D metabolism changed in a similar way between groups: 3-epi-25(OH)D_3_ levels increased until the Day 3, after which they decreased by the Day 7; 24,25(OH)_2_D_3_ levels showed more graduate elevation throughout the follow-up. In both groups 25(OH)D_3_/24,25(OH)_2_D_3_ ratios increased by Day 1, after which they decreased by Day 7, and 25(OH)D_3_/1,25(OH)_2_D_3_ ratios increased by Day 1, after which they remained stable. DBP levels didn’t change and free 25(OH)D levels showed an increase in both groups during the follow-up. The levels of 25(OH)D_2_ did not exceed 0.5 ng/mL in all examined individuals throughout the study. Among assessed parameters of vitamin D metabolism, higher 25(OH)D_3_/24,25(OH)_2_D_3_ ratios in the study group was the only difference between the groups which remained significant throughout the observation period (*p* < 0.05) (Figure 2). 

## 4. Discussion

The main goal of our study was to evaluate the 25(OH)D_3_ levels and its response to the therapeutic dose of cholecalciferol in patients with CD as compared to healthy individuals. We observed no difference in baseline 25(OH)D_3_ assessed by UPLC-MS/MS between groups. Similar to our data were obtained in most studies conducted specifically in the state of endogenous hypercortisolism in humans [12,15] and dogs [14]. The study by Kugai et al. lacked control group and reported plasma levels of 25(OH)D corresponding to the vitamin D deficiency in most of the examined patients [10], while in our study only 2/3 of the patients with CD had 25(OH)D levels below 20 ng/mL. As for exogenous hypercortisolism, the meta-analysis aimed to explore serum 25(OH)D levels in glucocorticoid users showed lower levels than in healthy controls, but similar to steroid-naive disease controls, thus causing concern regarding the influence of the disease status on 25(OH)D levels [24]. Somewhat surprisingly, we obtained significantly discordant results in the study group when screening total 25(OH)D by ELISA and when measuring baseline 25(OH)D_3_ by UPLC-MS/MS, since the initial difference between the groups revealed by ELISA data with lower total 25(OH)D levels in the study group was not replicated by UPLC-MS/MS. It should be noted that our ELISA method did not participate in an external quality control program at the time of the study unlike UPLC-MS/MS; furthermore, a lower analytical performance was previously described for this technique with tendency for low specificity and lower measurement results [45]. 

When assessing other parameters of vitamin D metabolism, the most significant finding was the higher 25(OH)D_3_/24,25(OH)_2_D_3_ ratio in CD patients, both initially and during the observation after the intake of the cholecalciferol loading dose, indicating consistently reduced activity of 24-hydroxylase, the main enzyme of vitamin D catabolism. Earlier clinical and experimental studies also suggested altered activity of enzymes of vitamin D metabolism in hypercortisolism. However, these studies were heterogeneous and aimed predominantly at studying the activity of 1α-hydroxylase [7,8,10,11,12,14], which was not altered in patients with CD as compared to healthy individuals in our study. In the setting of the short-term glucocorticoid administration, Lindgren et al. showed transient increase in 24,25(OH)_2_D_3_ levels in rats [8], while in the study of Hahn et al. there was no change in 24,25(OH)_2_D_3_ levels [11]. Dogs with CD had similar 24,25(OH)_2_D_3_ levels before and after hypophysectomy as well as compared to control dogs [14]. The only study of considerably similar design by Kugai et al. reported low-normal 24,25(OH)_2_D_3_ in patients with Cushing’s syndrome [10], which is consistent with our result, as well as some experimental works indicative of suppression on CYP24A1 expression by glucocorticoids in human osteoblasts [23], liver and intestine [21] and in rat brain and myocardium [22]. However, in the present work, the activity of 24-hydroxylase in patients with hypercortisolism was for the first time evaluated by calculating the 25(OH)D_3_/24,25(OH)_2_D_3_ ratio, which has recently emerged as a new tool for vitamin D status assessment [46,47]. Given the correlation of this parameter with laboratory marker of the underlying disease activity (24-h UFC), a direct effect of cortisol overproduction on 24-hydroxylase activity might be assumed. Interestingly, it seems that the decreased activity of 24-hydroxylase observed in CD influenced the effectiveness of cholecalciferol treatment, decreasing the negative effect of obesity, as patients with CD had similar increase in 25(OH)D_3_ in obese and non-obese state and lacked correlation between Δ25(OH)D_3_ and BMI, as opposed to the control group. Moreover, the increase in 25(OH)D_3_ in obese patients from the control group was lower not only than in non-obese controls, but also than in obese patients with CD. 

Another intriguing finding was lower levels of free 25(OH)D observed in patients with CD despite similar DBP levels and lower albumin levels, which, on the contrary, allows one to expect higher values of free 25(OH)D. Considering the weaker correlation between the measured and calculated free 25(OH)D in patients with CD, as well as the lack of correlation of the measured 25(OH)D with the main transport protein, an altered affinity of DBP might be suspected. One possible explanation is protein glycosylation as a consequence of diabetes mellitus, which was present in half of the patients [38,48,49]. After cholecalciferol intake, which was accompanied by an increase in free 25(OH)D, the differences between the groups were leveled; therefore, another suggested explanation might be competitive binding to the ligand. Since actin binds DBP with high affinity [50] and considering catabolic action of glucocorticoids on muscle tissue [51], actin is a presumable competing ligand candidate. Although this is mostly speculative, as far as the authors are aware, the present work was the first to assess free vitamin D in the glucocorticoid excess, so the described findings require verification of reproducibility and further evaluation.

The obtained discrepancies in the biochemical parameters characterizing calcium and phosphorus metabolism were generally consistent with the data of early studies discussed in the introduction [12,25,26,27,28,29], except for similar to controls serum phosphorus levels and lower prevalence of hypercalciuria. An interesting observation was the complete absence of the PTH decrease in patients with CD after receiving a loading dose of cholecalciferol. The mechanism of this phenomenon is not entirely clear, we tend to agree with the earlier hypothesis that this may be an adaptation to chronic urinary calcium loss [52].

Our research is distinguished by a number of important strengths: a prospective design, substantial sample of patients with CD, accounting for social and behavioral factors affecting vitamin levels D, comprehensive spectrum of vitamin D metabolism parameters investigated and participation in an external quality control program for vitamin D metabolites measurement.

Nevertheless, the study also had several limitations: the amount of dietary vitamin D and phosphorus, as well as possible differences in DBP affinity to vitamin D metabolites due to genetic isoforms of DBP [53] or other possible involved parameters (e.g., fibroblast growth factor-23) were not taken into account. A few patients from both groups received therapy with possible impact on vitamin D and calcium metabolism within 3 months preceding the participation in the study (spironolactone, diuretics, proton pump inhibitors, oral contraceptives, antifungal treatment, antidepressants, barbiturates, antiepileptic drugs). The groups had a trend for differences in sex and BMI (*p* = 0.07 for both parameters). Also, the study lacked a study group of patients with remission of CD to test the hypotheses put forward, however, this is a promising direction for further research.

## 5. Conclusions

We report that patients with endogenous ACTH-dependent hypercortisolism of pituitary origin have a consistently higher 25(OH)D_3_/24,25(OH)_2_D_3_ ratio than healthy controls, which is indicative of a decrease in 24-hydroxylase activity. This altered activity of the principal vitamin D catabolism might influence the effectiveness of cholecalciferol treatment. There is also a lack of clarity regarding the lower levels of free 25(OH)D observed in patients with CD, which require further study. To test the proposed hypotheses and to develop specialized clinical guidelines for these patients, longer-term randomized clinical trials are needed.

## Figures and Tables

**Table 1:** General characteristics of the patients at the baseline visits. For detailed description of the data format please refer to the Section 2.

Parameter	Study Group (*n* = 30)	Control Group (*n* = 30)	*p*
Age, years	39.1 [31.2; 48.2]	33.4 [26.5; 42.5]	0.12
Sex (female/male, *n*)	26/4	19/11	0.07
BMI, kg/m^2^	30.9 [27.1; 31.6]	27.2 [25.4; 30.4]	0.07
25(OH)D total, ng/mL	13.1 [9.6; 17.9]	21.7 [14.4; 28.0]	0.002

**Table 2:** Characteristics of the patients with Cushing’s disease (CD) in terms of the underlying disease.

Parameter	Value
24-h UFC, nmoL/24 h	1227 [813; 2970]
Morning ACTH, pg/mL	87 [60; 125]
Diabetes mellitus, *n* (%)	15 (50%)
HbA1c, %	7.8 [7.0; 8.4]
History of low energy fracture, *n* (%)	7 (23%)

**Table 3:** Questionnaire results.

Parameter	Study Group (*n* = 30)	Control Group (*n* = 30)	*p*
Current smokers, *n* (%)	6 (20%)	13 (43%)	0.09
Total smokers, *n* (%)	10 (33%)	18 (60%)	0.07
Alcohol units, per week	0 [0; 0]	1 [0; 2]	0.007
Exercises lasting more than 30 min, per week	5 [2; 7]	3 [2; 3]	0.09
Dairy products consumption, servings per day	1 [1; 1]	1 [1; 1]	1.0
Meat dishes consumption, portions per week	5 [4; 7]	5 [3; 7]	0.64
Coffee consumption, cups per week	6 [2; 8]	7 [1; 10]	0.4
Soft drinks, mL per week	0 [0; 0]	0 [0; 0]	0.76
Travelers to the south, *n* (%)	3 (10%)	4 (13%)	1.0
Daytime walks in the sunny weather, *n*	7 [0; 20]	4 [1; 11]	0.49
Solarium usage, *n* (%)	0	1 (3%)	1.0

**Table 4:** Changes in the levels of the biochemical parameters and parathyroid hormone (PTH) during the study.

Laboratory Parameter	Group	Day 0	Day 1	Day 3	Day 7	*p* (Friedman ANOVA)	*p* (Day 0–1)	*p* (Day 1–3)	*p* (Day 3–7)	Reference Range
Total calcium, mmol/L	Study	2.39 [2.25; 2.44]	2.37 [2.31; 2.47]	2.35 [2.27; 2.46]	2.39 [2.27; 2.51]	0.89	-	-	-	2.15–2.55
	Control	2.37 [2.31; 2.43]	2.41 [2.36; 2.46]	2.41 [2.37; 2.46]	2.37 [2.34; 2.48]	0.03	0.01	0.47	0.28	
Albumin-adjusted calcium, mmol/L	Study	2.28 [2.21; 2.36]	2.31 [2.23; 2.38]	2.30 [2.22; 2.36]	2.31 [2.23; 2.37]	0.92	-	-	-	2.15–2.55
	Control	2.25 [2.21; 2.31]	2.29 [2.23; 2.33]	2.30 [2.26; 2.35]	2.27 [2.24; 2.34]	0.005	0.01	0.24	0.29	
Phosphorus, mmol/L	Study	1.04 [0.98; 1.13]	1.15 [1.02; 1.19]	1.18 [1.04; 1.23]	1.07 [0.97; 1.19]	0.003	0.006	0.58	0.03	0.74–1.52
	Control	1.10 [1.00; 1.22]	1.15 [1.01; 1.26]	1.19 [1.07; 1.27]	1.15 [1.09; 1.31]	0.06	-	-	-	
PTH, pg/mL	Study	38.9 [33.8; 55.2]	39.5 [29.7; 52.3]	40.1 [31.7; 52.8]	40.1 [30.5; 53.6]	0.6	-	-	-	15–65
	Control	38.6 [31.0; 50.3]	37.0 [28.9; 51.4]	35.8 [28.9; 45.3]	34.3 [25.3; 47.7]	0.03	0.74	0.02	0.93	
Creatinine, μmol/L	Study	67.6 [62.4; 70.1] *	68.9 [63.4; 72.8] *	68.9 [62.1; 72.9]	69.0 [63.3; 72.5]	0.3	-	-	-	63–110 (male) 50–98 (female)
	Control	70.3 [67.4; 78.0]	73.5 [66.9; 79.8]	70.3 [67.1; 79.4]	72.2 [64.0; 83.9]	0.02	0.002	0.20	0.21	
Albumin, g/L	Study	44 [42; 46] *	44 [41; 45] *	44 [40; 47] *	44 [41; 47]	0.3	-	-	-	35–50
	Control	46 [44; 47]	46 [44; 48]	46 [44; 47]	46 [44; 47]	0.48	-	-	-	
Magnesium, mmol/L	Study	0.87 [0.81; 0.92] *	0.86 [0.79; 0.94] *	0.84 [0.78; 0.91] *	0.87 [0.81; 0.93] *	0.27	-	-	-	0.7–1.05
	Control	0.82 [0.76; 0.85]	0.79 [0.77; 0.84]	0.79 [0.76; 0.82]	0.79 [0.75; 0.84]	0.67	-	-	-	
Urine calcium-creatinine ratio, mmol/mmol	Study	0.36 [0.16; 0.49]	0.49 [0.28; 0.63] *	0.37 [0.22; 0.59]	0.49 [0.23; 0.80]	0.05	-	-	-	0.1–0.8
	Control	0.30 [0.13; 0.42]	0.26 [0.21; 0.41]	0.29 [0.21; 0.42]	0.35 [0.20; 0.50]	0.88	-	-	-	
Urine phosphorus-creatinine ratio, mmol/mmol	Study	2.6 [2.0; 3.1] *	2.6 [1.7; 3.1] *	2.6 [2.0; 3.6] *	3.4 [2.4; 4.1] *	0.001	0.51	0.66	0.02	1.4–3.5
	Control	1.8 [1.4; 2.7]	1.7 [0.9; 2.4]	1.6 [1.4; 2.3]	1.7 [1.2; 2.3]	0.09	-	-	-	

* Significant difference in between-group comparison.

**Table 5:** Changes in the levels of free 25(OH)D, vitamin D-binding protein (DBP) and vitamin D metabolites during the study.

Laboratory Parameter	Group	Day 0	Day 1	Day 3	Day 7	*p* (Friedman ANOVA)	*p* (Day 0–1)	*p* (Day 1–3)	*p* (Day 3–7)	Reference Range
Free 25(OH)D, pg/mL	Study	4.9 [4.0; 6.1] *	10.7 [8.4; 12.5]	12.9 [11.0; 14.3]	11.4 [10.0; 12.5]	<0.001	<0.001	<0.001	<0.001	2.4–35 ^1^
	Control	6.4 [4.1; 7.7]	12.1 [9.5; 15.0]	14.0 [10.3; 18.1]	12.9 [9.4; 15.4]	<0.001	<0.001	<0.001	<0.001	
DBP, mg/L	Study	270 [227; 298]	277 [247; 328]	276 [236; 301] *	252 [206; 281]	0.16	-	-	-	176–623 ^1^
	Control	247 [212; 281]	258 [237; 300]	236 [204; 274]	245 [220; 277]	0.31	-	-	-	
25(OH)D_3_, ng/mL	Study	17.9 [13.0; 24.5]	34.5 [27.6; 38.8]	37.6 [33.2; 45.5]	35.4 [32.1; 42.7]	<0.001	<0.001	<0.001	0.01	≥30 ^2^
	Control	19.5 [12.5; 25.7]	31.0 [28.1; 35.0]	33.9 [30.2; 43.1]	34.3 [30.9; 42.9]	<0.001	<0.001	<0.001	0.65	
3-epi-25(OH)D_3_, ng/mL	Study	0.8 [0.6; 1.1] *	3.0 [2.4; 3.5]	4.2 [3.6; 5.1]	3.1 [2.7; 3.8]	<0.001	<0.001	<0.001	<0.001	not available
	Control	1.4 [0.9; 1.7]	2.7 [2.1; 3.5]	3.9 [3.3; 4.9]	3.6 [3.0; 4.6]	<0.001	<0.001	<0.001	0.003	
1,25(OH)_2_D_3_, pg/mL	Study	41 [35; 50]	48 [37; 53]	47 [42; 56]	42 [39; 52]	<0.001	<0.001	0.22	0.02	25–66 ^3^
	Control	42 [34; 48]	48 [41; 55]	46 [39; 54]	43 [35; 47]	0.09	-	-	-	
24,25(OH)_2_D_3_, ng/mL	Study	1.0 [0.5; 1.4] *	1.4 [0.8; 1.8] *	2.1 [1.6; 2.8]	2.6 [1.8; 3.1]	<0.001	<0.001	<0.001	<0.001	0.5–5.6 ^3^
	Control	1.5 [0.9; 2.6]	1.8 [1.2; 2.6]	3.0 [1.9; 3.6]	3.2 [2.1; 4.2]	<0.001	<0.001	<0.001	<0.001	
25(OH)D_3_/24,25(OH)_2_D_3_	Study	19.5 [15.3; 26.8] *	26.8 [21.2; 32.5] *	17.4 [15.9; 21.0] *	13.9 [12.3; 18.8] *	<0.001	<0.001	<0.001	<0.001	7–23 ^3^
	Control	12.7 [9.9; 17.0]	18.2 [14.2; 21.3]	13.1 [10.6; 18.2]	12.3 [9.0; 14.9]	<0.001	<0.001	<0.001	<0.001	
25(OH)D_3_/1,25(OH)_2_D_3_	Study	425 [373; 555]	716 [588; 917]	783 [628; 1034]	814 [666; 1044]	<0.001	<0.001	0.08	0.86	not available
	Control	501 [356; 641]	689 [548; 789]	757 [602; 972]	851 [727; 1028]	<0.001	<0.001	0.03	0.05	

* Significant difference in between-group comparison. ^1^ Reference ranges are specified according to kit manufacturers’ recommendations. ^2^ Reference range is given for total 25(OH)D according to the clinical guidelines [34,42]; the 25(OH)D_2_ fraction is negligible (<0.5 ng/mL in absolute values) for the purposes of this study. ^3^ Reference ranges are given according to the literature data [43,44].

## Data Availability

The datasets generated during and/or analyzed during the current study are available from the corresponding author on reasonable request.

## Supplementary Materials

The following are available online at https://www.mdpi.com/article/10.3390/nu13124329/s1, Method validation against DEQAS, Figure S1: Comparison between DEQAS data for 25(OH)D scheme and our lab results, Figure S2: Comparison between DEQAS data for 1,25(OH)_2_D scheme and our lab results.
