# Supplementary material for: Assessment of Vitamin D Metabolism in Patients with Cushing’s Disease in Response to 150,000 IU Cholecalciferol Treatment
Source: Nutrients. 2021 Nov 30;13(12):4329. doi: 10.3390/nu13124329 (PMC8704048; doi:10.3390/nu13124329)
Supplement: Supplementary file 1 [file nutrients-13-04329-s001.zip › nutrients-1483833-supplementary.pdf]

SUPPORTING INFORMATION

Method validation against DEQAS

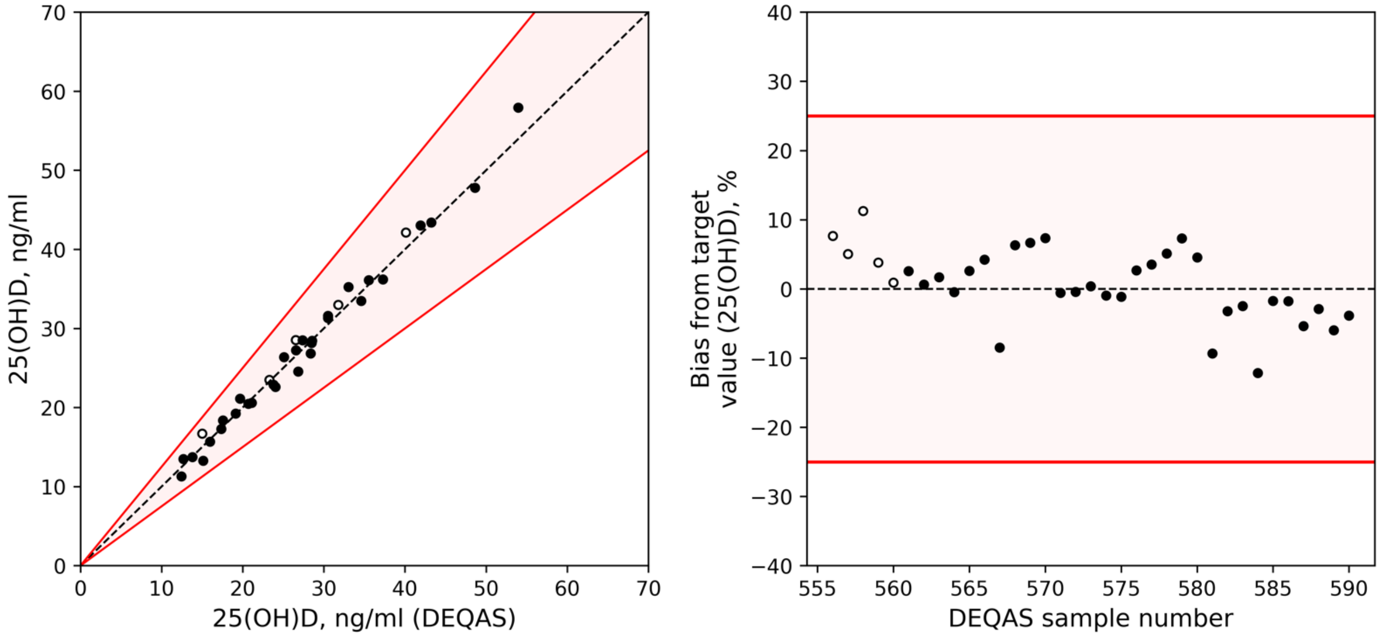

Figure S1 *Comparison between DEQAS data for 25(OH)D scheme and our lab results. Solid dotes denote blind results submitted to DEQAS as lab 2388 prior to publication of the report. Red area indicates DEQAS acceptable range (±25% from the target value)*

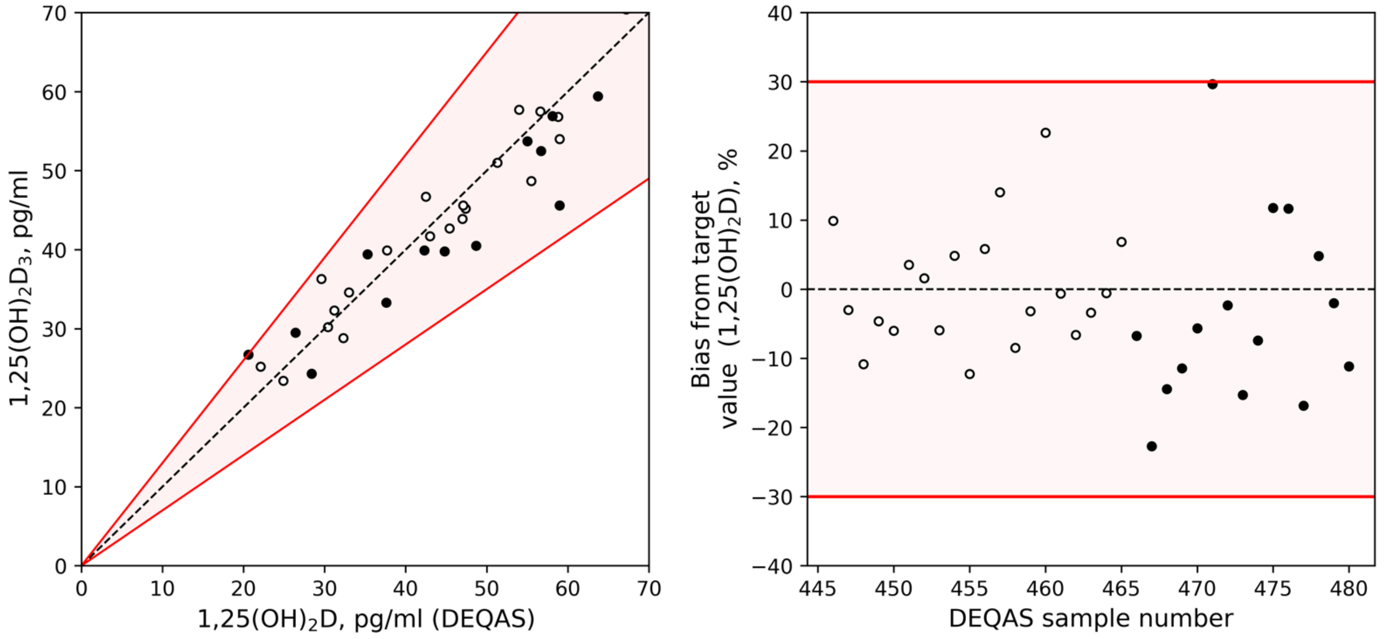

Figure S2 *Comparison between DEQAS data for 1,25(OH)$_2$D scheme and our lab results. Solid dotes denote blind results submitted to DEQAS as lab 2388 prior to publication of the report. Red area indicates DEQAS acceptable range (±30% from the target value)*
